# Dental Reunion

**Published:** 1873-05

**Authors:** 


### DENTAL REUNION.

### FOURTH ANNUAL MEETING OF THE SECOND DISTRICT DENTAL SOCIETY OF NEW YORK.

The fourth annual meeting of the society was held Tuesday April 8, at the residence of Dr. W. G. Mirick, No. 156 Clinton street, Brooklyn. There was a large gathering of distinguished members of the dental profession present.

This society embraces nine counties, and is at present in a most flourishing condition. Its reunions are regarded with great interest. In addition to all the more prominent local dentists, many leading representatives of the profession from various parts of the country were present, among whom were the following: Dr. Atkinson, New York; Dr. Jarvis, New York; Professor Shepard, Harvard University; Dr. Kingsley, Dr. S. S. White, Philadelphia; Dr. Wheeler, Albany; Dr. N. W. Hawes, Boston; Professor McQuillan, Philadelphia; Professor Woodward, New York; Dr. Ambler, New York; Dr. Comlie, Mexico; Dr. A. C. Hawes, New York; Dr. Miller, New York; Dr. Brunson, New York; Dr. Bogue New York; Professor Francis Abbott, New York; Dr., Clowes, New York; Dr. Barry, New York; Dr. Carr, New York; and many others.

The meeting was called to order at 3 o'clock. Dr. O. E. Hill, the President of the Society, in the Chair, and Dr. M. E. Elmendorf, Secretary.

After the reading of the minutes of the preceding meeting, and the transation of some formal business,

### THE ELECTION OF OFFICERS

for the ensuing year was taken up. The vote was by ballot. The following is the result of the vote:

President—Dr. W. S. Elliott.

Vice-President—Dr. William Jarvie.

Secretary—Dr. M. E. Elmendorf.

Corresponding Secretary—Dr. F. W. Dolbear.

Treasurer—Dr. Mirick.

Librarian—Dr. O. E. Hill.

Censors—Drs. Straw, Marvin, Parks, Jarvie and Hurd.

Delegates to State Society—Dr. O. E. Hill and Dr. A. H. Brockway.

Article 3, Chapter 1, of the By-laws was then, on motion, amended to read as follows:

"Our annual meeting shall be held on the first Monday in April of every year, and quarterly meetings on the first Monday in January, July and October."

### TREASURER'S REPORT.

Dr. Mirick, the Treasurer, submitted the following report:

| | |
|---|---|
| Balance last report. .. .. .. .. .. .. .. | $256,69 |
| Annual dues. .. .. .. .. .. .. .. .. | 132,00 |
| Interest etc. .. .. .. .. .. .. .. .. | 18,77 |
| | 407,46 |
| Expenditures. .. .. .. .. .. | 210,77 |
| Balance on hand | 196,69 |

Dr. Dolbear, in his report as Corresponding Secretary, referred to the great interest which has been taken in the society during the past year. He has ascertained that there are 228 resident dentists in this judicial district.

### INCIDENTS OF OFFICE PRACTICE.

Dr. Jarvie, Chairman of the Committee of Arragements, then read several communications from dentists who were from various causes prevented from attending. These letters, in almost every instance, contained some interesting "incident of office practice," that being the subject set down for discussion.

Dr. N. C. Barrett, of Utica, in his communication, referred to "facial neuralgia," and strongly recommended the use of Misereum.

Dr. Allport of Chicago, wrote to his brethren, and earnestly recommended them to abandon the use of highly cohesive gold and heavy foil.

At the request of the chairman, an animated discussion followed in reference to the subjects suggested by the communications referred to.

Dr. Atkinson took exception to Dr. Barrett's views in reference to the cure of neuralgia, and to Dr. Chase's in reference to the use of heavy foils.

Dr. N. W. Hawes, of Harvard University was next introduced, and read a brief but very able and concise paper, entitled "The reflex influence of Female Diseases upon the teeth, and the reflex influence of the teeth upon other organs of the system." Dr. Hawes called attention to the great sympathy which exists between different organs of the body, and how much the health of the offspring depends on that of the parents, and especially of the mother. He cited several forcible illustrations in support of his views.

Dr. Hawes' paper was listened to with great attention, and evidently produced a strong impression on those present.

Dr. Jarvis, speaking of the "Preservation of the Teeth," said that a certain amount of operating was necessary, but he had learned by twenty-five years' practice that the teeth could be preserved, first, by securing the proper condition, and then keeping the proper condition. There will then be no necessity for restoring them. The plan will be less laborious, less painful, and nobler than the constant "tinkering at the little holes."

Dr. Truman, referring to Dr. Hawes' paper, spoke of the necessity of dentists being well posted in collateral subjects. He had entire faith in the preservative tendency of cohesive foil. He disapproved of the practice of filling children's teeth with metal before they reached the age of fifteen years.

Dr. Shannon agreed with Dr. Truman on the latter subject, but differed with him in reference to hard foil, which he did not consider as preservative as soft foil.

Dr. Kingsley explained his system of using foils, and stated that he did not think there would be so much difference of opinion if the speakers really understood what they meant by hard, soft, adhesive and cohesive foils.

Dr. Shepard, of Boston, made an interesting address. He did not think it possible to educate people up to that point that they would keep their mouth in such a condition as to secure the teeth against disease. He recommended the use of a single strand of floss silk as a ligature.

Dr. Atkinson, Dr. Merrick, Dr. Hawes, Professor McQuillan, Dr. Marvin, Dr. Kingsley, and others, took part in the discussion, which was not brought to a close till nearly seven o'clock.

THE RUBBER LICENSE QUESTION.

Dr. Marvin presented a long preamble and resolutions bearing on the rubber license question and the Gardner appeal case.

After denouncing in bitter terms the alleged double-dealing of General John A. Foster, of New York, at one time counsel for the Dental Society; the resolution concludes with a warm eulogy on Dr. S. S. White, who has represented the dentists of the United States in the great legal struggle. The resolution was carried with great enthusiasm.

Dr. White followed with a few remarks on the rubber patent question. He believed the patent was invalid, and hoped to have it so declared.

Newburg having been selected as the next place of meeting, an adjournment took place.

